# Dietary flavonoid intake and risk of incident depression in midlife and older women^1^,^2^,^3^

**DOI:** 10.3945/ajcn.115.124545

**Published:** 2016-07-13

**Authors:** Shun-Chiao Chang, Aedin Cassidy, Walter C Willett, Eric B Rimm, Eilis J O’Reilly, Olivia I Okereke

**Affiliations:** 4 Channing Division of Network Medicine, Department of Medicine, and; 5 Department of Psychiatry, Brigham and Women’s Hospital and Harvard Medical School, Boston, MA;; 6 Department of Nutrition, Norwich Medical School, University of East Anglia, Norwich, United Kingdom; and; 7 Departments of Nutrition and; 8 Epidemiology, Harvard T.H. Chan School of Public Health, Boston, MA

**Keywords:** depression, epidemiology, flavonoids, geriatrics, Nurses’ Health Study, prospective cohort

## Abstract

**Background:** The impact of dietary flavonoid intakes on risk of depression is unclear.

**Objective:** We prospectively examined associations between estimated habitual intakes of dietary flavonoids and depression risk.

**Design:** We followed 82,643 women without a previous history of depression at baseline from the Nurses’ Health Study [(NHS) aged 53–80 y] and the Nurses’ Health Study II [(NHSII) aged 36–55 y]. Intakes of total flavonoids and subclasses (flavonols, flavones, flavanones, anthocyanins, flavan-3-ols, polymeric flavonoids, and proanthocyanidins) were calculated from validated food-frequency questionnaires collected every 2–4 y. Depression was defined as physician- or clinician-diagnosed depression or antidepressant use and was self-reported in response to periodic questionnaires. Cox proportional hazards models were performed to examine associations.

**Results:** A total of 10,752 incident depression cases occurred during a 10-y follow-up. Inverse associations between flavonol, flavone, and flavanone intakes and depression risk were observed. Pooled multivariable-adjusted HRs (95% CIs) were 0.93 (0.88, 0.99), 0.92 (0.86, 0.98), and 0.90 (0.85, 0.96) when comparing the highest (quintile 5) with the lowest (quintile 1) quintiles, respectively, with evidence of linear trends across quintiles (*P*-trend = 0.0004–0.08). In flavonoid-rich food-based analyses, the HR was 0.82 (95% CI: 0.74, 0.91) among participants who consumed ≥2 servings citrus fruit or juices/d compared with <1 serving/wk. In the NHS only, total flavonoids, polymers, and proanthocyanidin intakes showed significantly (9–12%) lower depression risks. In analyses among late-life NHS participants (aged ≥65 y at baseline or during follow-up), for whom we were able to incorporate depressive symptoms into the outcome definition, higher intakes of all flavonoid subclasses except for flavan-3-ols were associated with significantly lower depression risk; flavones and proanthocyanidins showed the strongest associations (HR for both: 0.83; 95% CI: 0.77, 0.90).

**Conclusions:** Higher flavonoid intakes may be associated with lower depression risk, particularly among older women. Further studies are needed to confirm these associations.

See corresponding editorial on page 549.

## INTRODUCTION

Depression is a major contributor to the global burden of disease- and illness-related disability (1). Although many patients respond favorably to treatment, residual symptoms and dysfunction from depression are common, especially among older adults (2). Therefore, effective prevention strategies are critical for lowering the overall burden (3).

Dietary modification seems appealing for depression prevention (4), yet the existing literature with regard to diet-depression relations has often been hampered by key limitations, including lack of prospective design and insufficient assessment of exposure (5). Dietary flavonoids represent a diverse range of polyphenolic compounds that occur naturally in plant foods, and they are present in substantial amounts in commonly consumed fruit, vegetables, grains, herbs, and beverages. The structural complexity of flavonoids has led to their subclassification as flavonols, flavones, flavanones, flavan-3-ols (and their oligomers, proanthocyanidins), isoflavones, and anthocyanins. Flavonoid compounds have been shown to exert antineuroinflammatory properties (6) and to suppress neuronal apoptosis and stimulate prosurvival signaling cascades (7)—mechanisms that may be involved in depression pathophysiology. In addition, some flavonoids appear to improve vascular health principally via enhanced blood flow (8, 9), which may be relevant to late-life depression, in which vascular disease burden has been implicated as a major factor (10). Moreover, higher habitual intakes of flavonoids/flavonoid-rich foods have already been associated with lower risks of many health outcomes (11–17). However, whether flavonoids present in the habitual diet are associated with depression is unknown.

Therefore, in this study, we prospectively examined whether self-reported long-term dietary intakes of flavonoid subclasses, as well as specific flavonoid-rich foods, were related to lower depression incidence, which was ascertained by self-report of physician or clinician diagnosis of depression or antidepressant use, in 2 large female cohorts: the Nurses’ Health Study (NHS)^9^ and the Nurses’ Health Study II (NHSII). We further investigated the presence and magnitude of associations for flavonoids/flavonoid-rich foods on depression risk among late-life participants (aged ≥65 y at baseline or during follow-up). We hypothesized that higher intakes of flavonoids, particularly flavonones, anthocyanins, and proanthocyanidins (17, 18), would be associated with lower depression risk.

## METHODS

### The NHS and NHSII

The NHS began in 1976 when 121,700 US female nurses, aged 30–55 y, returned a mailed questionnaire on lifestyle and medical history. In 1989, the NHSII began when a younger cohort of 116,430 women, aged 25–42 y, was enrolled via responding to similar mailed questionnaires. Since the start of each cohort, participants have received questionnaires biennially to update medical outcomes and health and lifestyle factors.

### Assessment of dietary flavonoid intake

A semiquantitative food-frequency questionnaire (FFQ) was included in 1984 and 1986 and every 4 y thereafter in the NHS and every 4 y since 1991 in the NHSII. On the FFQs, participants were asked how often, on average, they consumed a standard portion of ∼130 different foods in the previous year.

A database for assessing intakes of different flavonoid subclasses was constructed as described elsewhere (11). Briefly, intakes of flavonoid-rich foods were converted into different flavonoid subclasses, by multiplying the consumption frequency of each food by its flavonoid content. Because flavonoid subclasses have structural variation and differ in both bioavailability and bioactivity, we focused on the main subclasses commonly consumed in the US diet: flavonols (quercetin, kaempferol, myricetin, and isohamnetin), flavones (luteolin and apigenin), flavanones (eriodictyol, hesperetin, and naringenin), flavan-3-ols monomers (catechins, gallocatechins, epicatechins, epigallocatechin, epicatechin-3-gallate, and epigallocatechin-3-gallate), anthocyanins (cyanidin, delphinidin, malvidin, pelargonin, petunidin, and peonidin), and polymers (proanthocyanidins, theaflavins, and thearubigins). We defined “total flavonoids” as the sum of these subclasses. In addition, we examined proanthocyanidins separately, summing monomers, dimers, trimers, 4- to 6-mers, 7- to 10-mers, and >10-mers, because of plausible specific benefits for neuroprotection (18). We estimated energy-adjusted cumulative average intakes to assess long-term flavonoid intake and to minimize within-person variation; intakes were categorized into cohort-specific quintiles for analyses. For questionnaire cycles without an FFQ, we used the cumulative average assessed through the previous cycle. The validity and reproducibility of the FFQs were previously reported: for example, correlations between several major dietary sources of flavonoids including apples, tea, and wine measured by weighted diet records and FFQs were 0.70, 0.77, and 0.83, respectively (19–21).

### Assessment of depression

Depression assessments in the NHS/NHSII include self-report of symptoms, medications, and diagnosis. Symptoms were first assessed by using the 5-item Mental Health Index (MHI-5) subscale of the Short-Form 36 Health Status Survey on the 1992, 1996, and 2000 questionnaires in the NHS and the 1993, 1997, and 2001 questionnaires in the NHSII. In the NHS, symptoms continued to be assessed by the following measures: the 10-item version of the Center for Epidemiologic Studies Depression (CESD-10) in 2004 and the 15-item version of the Geriatric Depression Scale (GDS-15) in 2008. The MHI-5, CESD-10, and GDS-15 all have published validated cutoffs for clinical depression (22–25). Questions on regular antidepressant use were assessed biennially since 1996 (NHS) or 1997 (NHSII). Finally, participants were first asked if they ever had doctor-diagnosed depression in 2000 (NHS) or 2001 (NHSII) and were assessed biennially afterward. Because these were the first years in which we could classify women as ever having physician- or clinician-diagnosed depression, we designated 2000 and 2001 as the study baselines for NHS and NHSII, respectively.

In both cohorts, incident depression was defined as the first occurrence of self-reported clinical indicators of depression (i.e., doctor-diagnosed depression and/or regular antidepressant use), as in previous work (26). For antidepressants, we included selective serotonin reuptake inhibitors (SSRIs) but excluded tricyclic antidepressants (TCAs), because evidence elsewhere indicated that TCAs would be more likely to be prescribed for other indications (27). In both cohorts, history of depression at baseline was determined by the following: *1*) self-report of ever physician-diagnosed depression, *2*) self-reported use of SSRIs, and/or *3*) MHI-5 score of ≤52 before or at baseline questionnaires.

In a secondary analysis we examined associations between flavonoid intakes and late-life depression risk (i.e., among women aged ≥65 y at baseline or during follow-up in the NHS; no women in the NHSII were in this age range during follow-up). Within this NHS subset, we incorporated the presence of severe depressive symptoms with the use of published cutoffs (≥10 on the CESD-10 and ≥6 on the GDS-15) in the case definition.

### Covariates

We selected sociodemographic, lifestyle, and health/medical factors a priori on the basis of previous literature on depression risk factors (28) and our own preliminary data on risk indicators of depression. Sociodemographic factors included age, census-tract median family income (a proxy of socioeconomic status), social network index (29), and subjective social status (10-point visual analog scale of subjective feeling about standing in US society). Lifestyle variables included cigarette smoking, BMI (in kg/m^2^), alcohol intake, coffee consumption (30), physical activity [determined by using a validated assessment from which metabolic equivalents (metabolic equivalent-hours per week) could be quantified] (31), and modified alternate Mediterranean diet (aMed) adherence (32) (excluding fruit, vegetable, and alcohol components to avoid overadjustment). Health/medical covariates included bodily pain, sleep problems (sleep difficulty and sleep duration), menopausal status, postmenopausal hormone use, multivitamin use, and medical comorbidities. Covariates were updated every 2–4 y, as available. We carried forward the covariate information in the previous questionnaire cycle if missing during follow-up.

### Sample for analysis

After the exclusion of participants with a missing baseline 2000 or 2001 questionnaire, implausible daily energy intake (<600 or >3500 kcal/d), missing information on previous history of depression, or no health examination anytime during the follow-up (i.e., no opportunity for depression detection by a clinician), 45,985 NHS and 36,658 NHSII women were included for analysis (**Supplemental Figure 1**). The institutional review board at Brigham and Women’s Hospital reviewed and approved this study, and participants provided informed consent by returning their questionnaires.

### Statistical analysis

Individuals contributed person-years from the baseline questionnaire return date to the date of incident depression, death, end of follow-up (NHS: June 2010; NHSII: June 2011), or last returned questionnaire, whichever occurred first. We used time-dependent Cox proportional hazards regressions to estimate the age- and multivariable-adjusted RRs of depression, measured by HRs with 95% CIs. Multivariable-adjusted models adjusted for the following: age, census-tract median family income (<$50,000, $50,000–69,999, or ≥$70,000/y), social network (quintiles), subjective social status [bottom 4 steps of the social ladder (i.e., lower status) or fifth step or above], cigarette smoking (never; past; or current:1–14, 15–24, or ≥25 cigarettes/d), BMI (continuous), alcohol intake (0, >0 to <5, 5 to <30, 30 to <45, or ≥45 g/d), physical activity (continuous), coffee consumption (≤1 serving/wk, 2–6 servings/wk, 1 serving/d, 2–3 servings/d, or ≥4 servings/d), modified aMed score (quintiles), total energy intake (kcal/d; continuous), current multivitamin use (yes or no), menopausal status and postmenopausal hormone use (premenopausal or postmenopausal; never, past, or current postmenopausal hormone user), sleep hours (≤6, 7–8, or ≥9 h/d), frequency of difficulty falling or staying asleep (none, little, or some to all of the time), bodily pain (none to mild or moderate to very severe), and medical comorbidities (hypertension, cardiovascular disease, diabetes, and hypercholesterolemia; yes or no).

In primary analyses, we first characterized flavonoid intakes as quintiles without assuming linear flavonoid-depression associations; we further tested for potential linear trends across flavonoid quintiles by using a continuous variable in which participants were assigned the median value of their quintile. We first conducted analyses separately for each cohort and then conducted meta-analyses by using random-effects models to obtain combined estimates. Because the follow-up began in 2000 (NHS) or 2001 (NHSII), but the closest information of dietary flavonoids was collected in 1998 (NHS) or 1999 (NHSII), the analyses featured a 2-y outcome lag, which mitigated potential reverse causation (e.g., incipient depression may lead to changes in diet).

To address concerns of exposure misclassification, outcome misclassification, or competing risks, we performed 3 sensitivity analyses. First, we stopped updating diet after a report of incident cancer (except for nonmelanoma skin cancer), cardiovascular disease, or diabetes, because individuals may substantially alter their diet after diagnosis of a major disease; instead, the cumulative averages of flavonoid intakes up to the most recent dietary data before diagnosis of a major disease were carried forward thereafter. Second, we applied alternative definitions of depression when examining flavonoid-depression associations—including definitions based on the presence of both diagnosis and antidepressant use (more strict definition), diagnosis only, or diagnosis or antidepressant use including the use of TCAs (less strict definition). Third, it is possible that individuals with severe chronic diseases (cancer, cardiovascular disease, or diabetes) were less likely to respond to the questionnaires; thus, we censored participants at the time when they first reported these chronic conditions.

In secondary analyses, we conducted food-based analyses. Specifically, we addressed foods that, on the basis of previous published work elsewhere (11), are known to contribute to the flavonoid subclasses that were associated with depression risk in our primary analyses; we related intake amounts of those foods to depression risk. In addition, we specifically investigated relations of flavonoids to risk of depression in late life in 41,920 NHS women aged ≥65 y at baseline or during follow-up.

All of the analyses were conducted with SAS version 9.3 (SAS Institute). All *P* values were 2-sided (*P* < 0.05).

## RESULTS

There were 10,752 cases (NHS: 4896; NHSII: 5856) of incident depression cases identified during follow-up. At baseline, participants with higher total flavonoid intakes had healthier lifestyle behaviors (including higher levels of physical activity and a lower prevalence of current smoking) and higher family income (**Table 1**). The polymer and flavone subclasses contributed most and least, respectively, to total flavonoid intakes (IQR: 98.7–272.9 compared with 1.1–2.6 mg/d). NHS and NHSII participants had high completion on exposure and outcome data collection during follow-up (**Supplemental Table 1**). During the 10-y follow-up, each participant, on average, contributed 8.73 person-years. The number of exposure and outcome data collections and the average follow-up years per person did not vary by quintiles of flavonoid intakes (data not shown).

**TABLE 1:** Age-standardized characteristics of the study population by quintiles of total flavonoid intake at baseline in the NHS (2000) and the NHSII (2001)^1^

	Quintile of total flavonoid intake					
	NHS (2000)			NHSII (2001)		
	1	3	5	1	3	5
*n*	9197	9197	9197	7331	7334	7332
Age, y	65.5 ± 7.0^2^	66.9 ± 7.0	66.8 ± 7.1	46.0 ± 4.7	46.1 ± 4.7	46.7 ± 4.5
BMI, kg/m^2^	26.6 ± 5.3	26.4 ± 4.9	26.3 ± 5.0	26.5 ± 6.2	25.8 ± 5.6	26.3 ± 5.9
Census-tract median family income, in 1000 US$	62.4 ± 23.6	64.4 ± 24.9	64.9 ± 24.9	64.4 ± 22.4	67.5 ± 24.9	66.1 ± 24.3
Total energy intake, kcal/d	1803.2 ± 458.9	1781.7 ± 436.2	1595.9 ± 393.6	1755.8 ± 467.7	1846.9 ± 458.5	1762.2 ± 462.4
Total flavonoids, mg/d	127.6 ± 30.0	266.2 ± 22.7	787.3 ± 396.4	126.0 ± 29.4	264.2 ± 22.7	779.4 ± 325.2
Flavonols, mg/d	9.9 ± 3.9	15.0 ± 4.1	28.3 ± 9.4	10.4 ± 4.8	15.9 ± 5.4	31.4 ± 11.3
Flavones, mg/d	1.4 ± 0.7	2.3 ± 0.9	2.2 ± 1.2	1.2 ± 0.8	2.0 ± 1.0	1.9 ± 1.1
Flavanones, mg/d	28.2 ± 18.6	49.8 ± 27.5	49.7 ± 33.2	21.9 ± 16.7	41.1 ± 28.4	38.4 ± 31.0
Flavan-3-ols, mg/d	12.3 ± 5.4	31.1 ± 11.0	137.1 ± 72.0	11.8 ± 5.2	29.7 ± 10.1	146.1 ± 75.7
Anthocyanins, mg/d	6.8 ± 4.5	13.8 ± 9.0	15.8 ± 15.8	6.7 ± 4.8	14.4 ± 9.4	15.9 ± 17.1
Polymeric flavonoids, mg/d	69.0 ± 21.5	154.2 ± 27.8	554.2 ± 324.7	75.3 ± 21.7	162.6 ± 27.2	548.8 ± 247.3
Proanthocyanidins, mg/d	65.9 ± 21.3	117.1 ± 33.5	169.5 ± 61.0	70.1 ± 22.1	123.7 ± 36.4	179.9 ± 65.0
Orange juice,^3^ servings/d	0.3 ± 0.3	0.5 ± 0.4	0.4 ± 0.4	0.2 ± 0.3	0.4 ± 0.4	0.3 ± 0.4
Oranges,^3^ servings/d	0.1 ± 0.1	0.3 ± 0.2	0.2 ± 0.2	0.1 ± 0.1	0.2 ± 0.2	0.2 ± 0.2
Grapefruit juice,^3^ servings/d	0.1 ± 0.1	0.1 ± 0.2	0.1 ± 0.2	0.0 ± 0.1	0.1 ± 0.2	0.1 ± 0.2
Grapefruit,^3^ servings/d	0.1 ± 0.1	0.2 ± 0.2	0.1 ± 0.2	0.0 ± 0.1	0.1 ± 0.1	0.1 ± 0.2
Apples or pears,^3^ servings/d	0.2 ± 0.1	0.4 ± 0.3	0.4 ± 0.3	0.1 ± 0.1	0.3 ± 0.2	0.3 ± 0.3
Tea,^3^ servings/d	0.1 ± 0.1	0.3 ± 0.3	1.8 ± 1.1	0.0 ± 0.1	0.2 ± 0.2	1.9 ± 1.0
Strawberries,^3^ servings/d	0.1 ± 0.1	0.1 ± 0.1	0.1 ± 0.1	0.1 ± 0.1	0.1 ± 0.1	0.1 ± 0.2
Blueberries,^3^ servings/d	0.0 ± 0.0	0.1 ± 0.1	0.1 ± 0.1	0.0 ± 0.0	0.1 ± 0.1	0.1 ± 0.1
Red wine,^3^ servings/d	0.0 ± 0.1	0.1 ± 0.2	0.1 ± 0.2	0.0 ± 0.1	0.1 ± 0.2	0.1 ± 0.2
Onions,^3^ servings/d	0.1 ± 0.2	0.2 ± 0.2	0.1 ± 0.2	0.1 ± 0.1	0.1 ± 0.2	0.1 ± 0.2
Coffee,^3^ servings/d	1.9 ± 1.5	1.5 ± 1.3	1.1 ± 1.1	1.3 ± 1.5	1.2 ± 1.2	0.9 ± 1.2
Alcohol, g/d	7.3 ± 11.0	5.7 ± 8.2	4.4 ± 6.9	3.5 ± 6.1	3.6 ± 5.4	3.1 ± 5.4
aMed score	3.6 ± 1.4	4.6 ± 1.4	4.1 ± 1.4	3.3 ± 1.5	4.6 ± 1.6	4.2 ± 1.6
Physical activity, MET-h/wk	14.8 ± 18.8	19.9 ± 22.9	18.2 ± 21.5	17.7 ± 21.4	23.8 ± 27.5	22.2 ± 25.5
Current smoker, %	13.3	5.0	6.4	9.8	4.7	6.2
Postmenopausal, %	98.9	98.9	98.7	30.2	30.1	32.3
Current hormone use,^4^ %	44.6	46.9	45.0	52.6	55.3	56.4
Low self-rated societal position (bottom 4 of 10 ladders), %	2.0	1.3	1.3	1.7	1.4	1.9
High social network (top 2 quintiles), %	29.7	33.5	30.8	32.2	35.3	34.9
Normal sleep hours (7–8 h/d), %	65.7	67.7	66.1	68.8	70.7	67.2
Difficulty falling or staying asleep, some to all of the time, %	32.5	30.3	29.7	23.8	23.5	23.5
Bodily pain, moderate or more, %	22.4	20.8	21.0	12.7	12.3	13.9
Current multivitamin use, %	63.9	69.6	67.5	53.2	61.5	59.7
Cancer, %	14.6	15.4	15.5	8.6	9.5	9.3
Hypertension, %	48.3	47.8	47.7	15.5	14.1	16.8
Hypercholesterolemia, %	60.2	60.1	60.7	28.8	26.4	27.5
Heart disease, %	8.0	6.5	7.9	1.1	0.9	0.8
Diabetes, %	8.2	7.4	7.9	2.2	2.1	2.1

1 *n* = 82,643. All variables were age-adjusted except for age. aMed, alternate Mediterranean diet; MET-h, metabolic equivalent task hours; NHS, Nurses’ Health Study; NHSII, Nurses’ Health Study II.

2 Mean ± SD (all such values).

3 One serving = 1 orange, one-half grapefruit, 1 small glass of orange juice, 1 small glass of grapefruit juice, 1 fresh apple or pear, 1 cup (240 mL) tea, 0.5 cup strawberries or blueberries, 0.5 cup onions, 4 oz (120 mL) glass red wine, or 1 cup caffeinated coffee.

4 Among postmenopausal women only.

**Table 2** shows the associations between cumulative averages of flavonoid intakes and depression risk in both cohorts. Observed incidence rates of depression were 12.3 and 18.2 cases/1000 person-years in the NHS and NHSII, respectively. In the multivariable-adjusted pooled analyses of all participants, individuals in the highest (quintile 5) compared with the lowest (quintile 1) quintile of flavonol, flavone, and flavanone intakes had a significant 7–10% reduction in depression risk; there was evidence of inverse linear trends across quintiles (*P*-trend = 0.08, 0.0004, and 0.0007, respectively) (Table 2). Results differed by cohort for other subclasses. Specifically, in the NHS, higher intakes of total polymers and proanthocyanidins were significantly associated with a lower risk of depression (*P*-trend = 0.04 and 0.03, respectively); yet, comparable results were not observed in the NHSII. Furthermore, a greater intake of total flavonoids was associated with significantly lower depression risk only in the NHS, with evidence of effect heterogeneity in several quintile groups between cohorts.

**TABLE 2:** HRs (95% CIs) for associations between flavonoid subclass intakes and incident depression among women in the NHS and the NHSII^1^

	Quintile of intake					
	1	2	3	4	5	*P*-trend
Total flavonoids, mg/d						
NHS	≤177.8	>177.8–238.9	>238.9–315.2	>315.2–462.5	>462.5	
Cases/person-years, *n*/*n*	1089/79,907	963/79,893	955/79,785	959/79,792	930/79,913	
Model 1^2^	1.00 (referent)	0.89 (0.81, 0.97)	0.89 (0.82, 0.97)	0.89 (0.81, 0.97)	0.85 (0.78, 0.93)	0.01
Model 2^3^	1.00 (referent)	0.91 (0.83, 1.00)	0.92 (0.84, 1.01)	0.91 (0.83, 1.00)	0.88 (0.80, 0.97)	0.04
NHSII	≤182.4	>182.4–247.0	>247.0–326.4	>326.4–478.7	>478.7	
Cases/person-years, *n*/*n*	1262/64,567	1236/64,393	1103/64,304	1096/64,361	1159/64,351	
Model 1^2^	1.00 (referent)	1.03 (0.95, 1.11)	0.95 (0.87, 1.03)	0.95 (0.87, 1.03)	0.98 (0.91, 1.06)	0.45
Model 2^3^	1.00 (referent)	1.03 (0.95, 1.11)	0.95 (0.88, 1.03)	0.96 (0.88, 1.04)	1.00 (0.92, 1.09)	0.91
Meta-analyzed results						
Random-effects model^4^	1.00 (referent)	0.97 (0.86, 1.09)	0.94 (0.88, 1.00)	0.94 (0.88, 1.00)	0.94 (0.84, 1.07)	0.30
* P*-heterogeneity		0.05	0.62	0.44	0.04	0.13
Flavonols, mg/d						
NHS	≤10.8	>10.8–13.8	>13.8–17.2	>17.2–22.5	>22.5	
Cases/person-years, *n*/*n*	1011/79,951	1044/79,876	995/79,773	949/79,803	897/79,888	
Model 1^2^	1.00 (referent)	1.05 (0.96, 1.15)	1.01 (0.93, 1.11)	0.97 (0.88, 1.06)	0.91 (0.83, 0.99)	0.003
Model 2^3^	1.00 (referent)	1.06 (0.97, 1.15)	1.03 (0.94, 1.12)	0.99 (0.90, 1.09)	0.93 (0.85, 1.02)	0.02
NHSII	≤11.3	>11.3–14.7	>14.7–18.7	>18.7–25.0	>25.0	
Cases/person-years, *n*/*n*	1253/64,658	1165/64,452	1139/64,441	1183/64,162	1116/64,263	
Model 1^2^	1.00 (referent)	0.98 (0.90, 1.06)	0.98 (0.90, 1.06)	1.04 (0.95, 1.12)	0.98 (0.90, 1.06)	0.96
Model 2^3^	1.00 (referent)	0.95 (0.87, 1.03)	0.93 (0.85, 1.01)	0.98 (0.90, 1.07)	0.94 (0.86, 1.02)	0.37
Meta-analyzed results						
Random-effects model^4^	1.00 (referent)	1.00 (0.90, 1.11)	0.97 (0.88, 1.07)	0.99 (0.93, 1.05)	0.93 (0.88, 0.99)	0.08
* P*-heterogeneity		0.07	0.12	0.91	0.90	0.22
Flavones, mg/d						
NHS	≤1.3	>1.3–1.8	>1.8–2.3	>2.3–3.0	>3.0	
Cases/person-years, *n*/*n*	1030/79,857	1003/79,992	1014/79,844	915/79,876	934/79,721	
Model 1^2^	1.00 (referent)	0.98 (0.90, 1.07)	0.99 (0.90, 1.08)	0.90 (0.82, 0.98)	0.91 (0.83, 0.99)	0.01
Model 2^3^	1.00 (referent)	0.99 (0.91, 1.09)	1.01 (0.92, 1.11)	0.92 (0.84, 1.01)	0.94 (0.86, 1.03)	0.07
NHSII	≤1.1	>1.1–1.5	>1.5–2.0	>2.0–2.6	>2.6	
Cases/person-years, *n*/*n*	1261/64,547	1286/64,418	1206/64,318	1090/64,366	1013/64,328	
Model 1^2^	1.00 (referent)	1.05 (0.98, 1.14)	1.02 (0.94, 1.11)	0.92 (0.85, 1.00)	0.88 (0.81, 0.96)	<0.0001
Model 2^3^	1.00 (referent)	1.04 (0.96, 1.13)	1.01 (0.93, 1.09)	0.93 (0.85, 1.01)	0.90 (0.83, 0.99)	0.001
Meta-analyzed results						
Random-effects model^4^	1.00 (referent)	1.02 (0.96, 1.08)	1.01 (0.95, 1.07)	0.92 (0.87, 0.98)	0.92 (0.86, 0.98)	0.0004
* P*-heterogeneity		0.46	0.94	0.84	0.53	0.34
Flavanones, mg/d						
NHS	≤20.2	>20.2–33.2	>33.2–46.5	>46.5–64.2	>64.2	
Cases/person-years, *n*/*n*	1037/79,801	971/79,877	984/79,845	956/79,914	948/79,854	
Model 1^2^	1.00 (referent)	0.93 (0.85, 1.02)	0.94 (0.86, 1.02)	0.90 (0.82, 0.98)	0.86 (0.79, 0.94)	0.001
Model 2^3^	1.00 (referent)	0.95 (0.87, 1.04)	0.97 (0.89, 1.06)	0.94 (0.85, 1.02)	0.91 (0.82, 0.99)	0.04
NHSII	≤14.0	>14.0–22.8	>22.8–33.9	>33.9–51.5	>51.5	
Cases/person-years, *n*/*n*	1235/64,295	1225/64,266	1206/64,295	1126/64,463	1064/64,657	
Model 1^2^	1.00 (referent)	1.00 (0.92, 1.08)	0.98 (0.91, 1.07)	0.91 (0.84, 0.99)	0.84 (0.77, 0.91)	<0.0001
Model 2^3^	1.00 (referent)	1.00 (0.92, 1.08)	0.99 (0.92, 1.08)	0.95 (0.87, 1.03)	0.90 (0.83, 0.98)	0.006
Meta-analyzed results						
Random-effects model^4^	1.00 (referent)	0.98 (0.92, 1.04)	0.98 (0.92, 1.04)	0.94 (0.89, 1.00)	0.90 (0.85, 0.96)	0.0007
* P*-heterogeneity		0.46	0.78	0.85	0.97	0.60
Flavan-3-ols, mg/d						
NHS	≤15.7	>15.7–24.8	>24.8–39.7	>39.7–72.1	>72.1	
Cases/person-years, *n*/*n*	1062/79,920	981/79,817	963/79,778	925/79,871	965/79,905	
Model 1^2^	1.00 (referent)	0.94 (0.86, 1.03)	0.94 (0.86, 1.03)	0.89 (0.81, 0.97)	0.92 (0.84, 1.01)	0.12
Model 2^3^	1.00 (referent)	0.96 (0.88, 1.05)	0.96 (0.88, 1.05)	0.91 (0.83, 0.99)	0.94 (0.86, 1.03)	0.18
NHSII	≤16.0	>16.0–25.2	>25.2–39.9	>39.9–74.9	>74.9	
Cases/person-years, *n*/*n*	1220/64,631	1164/64,339	1132/64,278	1154/64,298	1186/64,431	
Model 1^2^	1.00 (referent)	1.01 (0.93, 1.09)	1.01 (0.93, 1.10)	1.02 (0.94, 1.11)	1.02 (0.95, 1.11)	0.56
Model 2^3^	1.00 (referent)	0.99 (0.91, 1.07)	0.97 (0.89, 1.05)	0.99 (0.91, 1.08)	1.00 (0.93, 1.09)	0.66
Meta-analyzed results						
Random-effects model^4^	1.00 (referent)	0.97 (0.92, 1.04)	0.97 (0.91, 1.03)	0.95 (0.87, 1.00)	0.97 (0.91, 1.04)	0.64
* P*-heterogeneity		0.72	0.93	0.15	0.27	0.19
Anthocyanins, mg/d						
NHS	≤5.9	>5.9–9.3	>9.3–13.4	>13.4–20.1	>20.1	
Cases/person-years, *n*/*n*	1099/79,947	983/79,946	942/79,821	910/79,880	962/79,696	
Model 1^2^	1.00 (referent)	0.91 (0.83, 0.99)	0.89 (0.81, 0.97)	0.87 (0.79, 0.95)	0.94 (0.86, 1.03)	0.38
Model 2^3^	1.00 (referent)	0.92 (0.85, 1.01)	0.92 (0.84, 1.00)	0.91 (0.83, 1.00)	1.00 (0.91, 1.10)	0.52
NHSII	≤6.3	>6.3–10.5	>10.5–15.3	>15.3–23.2	>23.2	
Cases/person-years, *n*/*n*	1331/64,672	1253/64,563	1154/64,355	1105/64,292	1013/64,095	
Model 1^2^	1.00 (referent)	1.00 (0.92, 1.08)	0.95 (0.88, 1.03)	0.95 (0.87, 1.02)	0.92 (0.84, 1.00)	0.02
Model 2^3^	1.00 (referent)	1.01 (0.93, 1.09)	0.97 (0.89, 1.05)	0.97 (0.89, 1.05)	0.95 (0.87, 1.04)	0.17
Meta-analyzed results						
Random-effects model^4^	1.00 (referent)	0.97 (0.89, 1.06)	0.94 (0.89, 1.00)	0.94 (0.88, 1.00)	0.97 (0.91, 1.04)	0.70
* P*-heterogeneity		0.14	0.38	0.33	0.41	0.17
Polymeric flavonoids, mg/d						
NHS	≤96.3	>96.3–137.2	>137.2–190.9	>190.9–300.6	>300.6	
Cases/person-years, *n*/*n*	1072/79,925	998/79,856	952/79,809	937/79,806	937/79,895	
Model 1^2^	1.00 (referent)	0.95 (0.87, 1.04)	0.92 (0.84, 1.00)	0.89 (0.82, 0.98)	0.89 (0.81, 0.97)	0.01
Model 2^3^	1.00 (referent)	0.98 (0.90, 1.07)	0.95 (0.87, 1.04)	0.93 (0.85, 1.02)	0.91 (0.83, 1.00)	0.04
NHSII	≤108.6	>108.6–151.9	>151.9–208.0	>208.0–321.9	>321.9	
Cases/person-years, *n*/*n*	1270/64,623	1181/64,379	1120/64,276	1124/64,321	1161/64,378	
Model 1^2^	1.00 (referent)	0.98 (0.90, 1.06)	0.96 (0.89, 1.05)	0.97 (0.89, 1.05)	0.98 (0.90, 1.06)	0.76
Model 2^3^	1.00 (referent)	0.97 (0.90, 1.05)	0.96 (0.89, 1.05)	0.97 (0.90, 1.06)	0.99 (0.91, 1.07)	0.91
Meta-analyzed results						
Random-effects model^4^	1.00 (referent)	0.98 (0.92, 1.04)	0.96 (0.90, 1.02)	0.95 (0.90, 1.01)	0.96 (0.88, 1.03)	0.39
* P*-heterogeneity		0.86	0.88	0.46	0.19	0.10
Proanthocyanidins, mg/d						
NHS	≤79.9	>79.9–103.3	>103.3–127.3	>127.3–160.0	>160.0	
Cases/person-years, *n*/*n*	1108/79,935	975/79,907	1002/79,877	929/79,775	882/79,796	
Model 1^2^	1.00 (referent)	0.89 (0.82, 0.98)	0.93 (0.85, 1.01)	0.87 (0.80, 0.95)	0.82 (0.75, 0.90)	<0.0001
Model 2^3^	1.00 (referent)	0.92 (0.84, 1.00)	0.96 (0.88, 1.05)	0.92 (0.84, 1.01)	0.89 (0.81, 0.97)	0.03
NHSII	≤87.7	>87.7–114.0	>114.0–141.5	>141.5–179.0	>179.0	
Cases/person-years, *n*/*n*	1298/64,671	1237/64,498	1170/64,384	1109/64,234	1042/64,189	
Model 1^2^	1.00 (referent)	1.00 (0.92, 1.08)	0.98 (0.91, 1.06)	0.95 (0.88, 1.03)	0.93 (0.86, 1.01)	0.05
Model 2^3^	1.00 (referent)	1.00 (0.93, 1.09)	0.99 (0.91, 1.07)	0.98 (0.90, 1.06)	0.97 (0.89, 1.06)	0.40
Meta-analyzed results						
Random-effects model^4^	1.00 (referent)	0.96 (0.88, 1.05)	0.97 (0.92, 1.03)	0.95 (0.89, 1.01)	0.93 (0.85, 1.02)	0.08
* P*-heterogeneity		0.13	0.61	0.39	0.17	0.24

1 *n* = 82,643. HRs (95% CIs) for depression by intake category of total flavonoids and individual subclasses of flavonoids were analyzed by using Cox proportional hazards models. The test for trend was performed by using the median value for each intake category. Depression was assessed by either self-reported physician diagnosis or regular use of antidepressant medication. NHS, Nurses’ Health Study; NHSII, Nurses’ Health Study II.

2 Model 1 adjusted for age and questionnaire cycle.

3 Model 2 adjusted for age, questionnaire cycle, total energy intake (continuous), social network (quintiles), alternate Mediterranean diet score (quintiles; excluding components of vegetables, fruit, and alcohol), census-tract family income (<$50,000, $50,000–69,999, or ≥$70,000/y), alcohol intake (0, 0.1–4.9, 5.0–29.9, 30–44.9, or ≥45.0 g/d), subjective self-rated societal position (low or other), cigarette smoking (never; past; current: 1–14, 15–24, or ≥25 cigarettes/d), BMI (continuous), physical activity in total metabolic equivalents per week (continuous), actual sleep hours (≤6, 7–8, or ≥9 h/d), frequency of difficulty falling or staying asleep (none, little, or some to all of the time), bodily pain (none to mild or moderate to very severe), current multivitamin use (yes or no), coffee consumption (≤1 serving/wk, 2–6 servings/wk, 1 serving/d, 2–3 servings/d, or ≥4 servings/d), menopausal status and postmenopausal hormone use [premenopausal or postmenopausal (never postmenopausal hormone user, past postmenopausal hormone user, or current postmenopausal hormone user)], history of hypertension (yes or no), history of cardiovascular disease (yes or no), history of diabetes (yes or no), and history of hypercholesterolemia (yes or no).

4 Data were meta-analyzed by using random-effects model of results from model 2.

A sensitivity analysis, in which dietary information was no longer updated after participants developed major chronic conditions, yielded results similar to those in Table 2 (**Supplemental Table 2**). Results were similar when different outcome definitions were applied, but the 95% CIs were wider when the more strict definition was used—likely due to the reduced numbers of cases (**Supplemental Table 3**). We conducted a competing risk analysis, in which participants were censored when a major disease (cancer, heart disease, or diabetes) occurred; this reduced person-years by 29% and 16% in the NHS and NHSII, respectively. However, findings from the competing risk analysis were similar to those from the primary analyses (**Supplemental Table 4**). Finally, given the potential for confounding by other nutrients in flavonoid-rich foods that may be associated with depression, we performed additional analyses that further adjusted for intakes of omega-3 fatty acids, vitamin C, vitamin B-6, vitamin B-12, and folate; the results were unchanged (data not shown). The results remained the same when the aMed score adjusted for in the model also included fruit and vegetable components (data not shown).

We then conducted food-based analyses for the major sources of the flavonoid subclasses that were significantly associated with lower depression risk in the pooled results in Table 2: flavonols, flavones, and flavanones. The predominant flavone and flavanone contributors, citrus (including orange and grapefruit) fruit and juices (33), were significantly inversely associated with depression risk in a dose-response fashion. Compared with citrus intake of <1 serving/wk, intakes of ≥2 servings/d were associated with an 18% reduction in depression risk (**Table 3**); this association was consistent in both cohorts. Both citrus fruit and juices were individually associated with lower depression risk (*P-*trend = 0.001 and 0.05, respectively); however, although moderate consumption of citrus fruit was related to lower depression risk, the significant association between citrus juice and lower depression risk was seen only at the highest consumption amount. With regard to flavonols, tea and onions are 2 major contributors. Being in the highest (≥4 cups/d) compared with the lowest (rarely or never) tea consumption group was significantly associated with a 12% lower risk of depression; onion intake was not associated with depression risk.

**TABLE 3:** Pooled HRs (95% CIs) for associations between intakes of flavonoid-rich food and incident depression among women in the NHS and the NHSII^1^

	Intake category^2^					
	1	2	3	4	5	*P*-trend
Citrus fruit and juice results	1.00 (referent)	0.94 (0.85, 1.05)	0.89 (0.78, 1.02)	0.85 (0.75, 0.97)	0.82 (0.74, 0.91)	<0.0001
Citrus fruit	1.00 (referent)	0.93 (0.88, 0.99)	0.91 (0.86, 0.96)	0.97 (0.83, 1.13)	0.87 (0.75, 1.01)	0.001
Citrus juice	1.00 (referent)	0.99 (0.88, 1.12)	0.98 (0.92, 1.05)	0.96 (0.88, 1.04)	0.90 (0.82, 0.98)	0.05
Tea	1.00 (referent)	0.96 (0.90, 1.02)	0.98 (0.92, 1.04)	0.96 (0.90, 1.03)	0.88 (0.78, 1.00)	0.30
Onions	1.00 (referent)	1.00 (0.94, 1.06)	0.98 (0.92, 1.05)	0.96 (0.89, 1.02)	0.99 (0.89, 1.09)	0.25

1 *n* = 82,643. Depression was assessed by either self-reported physician diagnosis or regular use of antidepressant medication. HRs (95% CIs) for depression by intake category of total flavonoids and individual subclasses of flavonoids were analyzed by using Cox proportional hazards models. The test for trend was performed by using the median value for each intake category. Model adjusted for age, questionnaire cycle, total energy intake (continuous), social network (quintiles), alternate Mediterranean diet score (quintiles), census-tract family income (<$50,000, $50,000–69,999, or ≥$70,000/y), alcohol intake (0, 0.1–4.9, 5.0–29.9, 30–44.9, or ≥45.0 g/d), subjective self-rated societal position (low or other), cigarette smoking (never; past; current: 1–14, 15–24, or ≥25 cigarettes/d), BMI (continuous), physical activity in total metabolic equivalents per week (continuous), actual sleep hours (≤6, 7–8, or ≥9 h/d), frequency of difficulty falling or staying asleep (none, little, or some to all of the time), bodily pain (none to mild or moderate to very severe), current multivitamin use (yes or no), coffee consumption (≤1 serving/wk, 2–6 servings/wk, 1 serving/d, 2–3 servings/d, or ≥4 servings/d), menopausal status and postmenopausal hormone use [premenopausal or postmenopausal (never postmenopausal hormone user, past postmenopausal hormone user, or current postmenopausal hormone user)], history of hypertension (yes or no), history of cardiovascular disease (yes or no), history of diabetes (yes or no), and history of hypercholesterolemia (yes or no). Data were meta-analyzed using random-effects models. NHS, Nurses’ Health Study; NHSII, Nurses’ Health Study II.

2 Category values were as follows—citrus fruit and juice, citrus fruit, and citrus juice: 1 (<1 serving/wk), 2 (1 serving/wk), 3 (2–6 servings/wk), 4 (1 serving/d), and 5 (≥2 servings/d); tea: 1 (<1 serving/mo), 2 (≥1 serving/mo to ≤1 serving/wk), 3 (2–6 servings/wk), 4 (1–3 servings/d), and 5 (≥4 servings/d); onion: 1 (<1 serving/mo), 2 (1–3 servings/mo), 3 (1 serving/wk), 4 (2–4 servings/wk), and 5 (≥5 servings/wk). One serving = 1 orange, one-half grapefruit, 1 small glass of orange juice, 1 small glass of grapefruit juice, 1 cup (240 mL) tea, or 0.5 cup onions.

In the analyses of flavonoids and late-life depression (i.e., women aged ≥65 y at baseline or during follow-up) (**Supplemental Table 5**), the observed incidence rate was 12.0 cases/1000 person-years when depression was defined by self-report of physician or clinician diagnosis or antidepressant treatment. When the outcome included diagnosis, antidepressant use, and/or clinically relevant depressive symptoms, the incidence rate was 24.2 cases/1000 person-years, which is consistent with sex-specific late-life incidence rates of any depression (i.e., either minor or major depression) observed elsewhere (34, 35). Importantly, comparing results by using the different definitions of depression, the patterns of inverse associations between dietary flavonoids and late-life depression were consistent. However, effect estimates appeared stronger, with narrower 95% CIs, in analyses that incorporated depressive symptoms. For example, placement in quintile 5 (compared with quintile 1) for almost all flavonoid subclasses was related to significantly lower depression risk, with evidence of linear trends. The exception was flavan-3-ols monomers (**Table 4**), which suggests that polymers of flavan-3-ols may be more relevant than monomers to late-life depression risk. The strongest associations were seen for flavones and proanthocyanidins (quintile 5 compared with quintile 1; HR for both: 0.83; 95% CI: 0.77, 0.90). The complementary food-based analysis showed that higher intakes of apples/pears and citrus were associated with lower depression risk (*P-*trend = 0.002 and 0.02, respectively) (**Table 5**).

**TABLE 4:** HRs (95% CIs) for associations between flavonoid subclass intakes and incident late-life depression among women in the NHS, aged ≥65 y^1^

	Quintile of intake					
	1	2	3	4	5	*P*-trend
Late-life depression, assessed by self-reported diagnosis, treatment, or severe symptoms^2^						
Total flavonoids	1.00 (referent)	0.96 (0.89, 1.03)	0.96 (0.89, 1.04)	0.92 (0.85, 0.99)	0.89 (0.82, 0.96)	0.003
Flavonols	1.00 (referent)	1.05 (0.97, 1.13)	0.98 (0.91, 1.06)	0.91 (0.84, 0.99)	0.90 (0.83, 0.97)	0.0001
Flavones	1.00 (referent)	0.96 (0.89, 1.04)	0.95 (0.88, 1.02)	0.91 (0.84, 0.98)	0.83 (0.77, 0.90)	<0.0001
Flavanones	1.00 (referent)	0.91 (0.84, 0.98)	0.96 (0.88, 1.03)	0.93 (0.86, 1.00)	0.86 (0.80, 0.93)	0.001
Flavan-3-ols	1.00 (referent)	0.97 (0.90, 1.05)	0.95 (0.88, 1.02)	0.90 (0.83, 0.97)	0.93 (0.86, 1.01)	0.07
Anthocyanins	1.00 (referent)	0.90 (0.84, 0.97)	0.92 (0.85, 0.99)	0.86 (0.79, 0.93)	0.90 (0.83, 0.97)	0.03
Polymeric flavonoids	1.00 (referent)	1.00 (0.92, 1.07)	0.96 (0.89, 1.03)	0.94 (0.87, 1.02)	0.88 (0.82, 0.96)	0.0007
Proanthocyanidins	1.00 (referent)	0.95 (0.88, 1.02)	0.96 (0.89, 1.03)	0.92 (0.85, 1.00)	0.83 (0.77, 0.90)	<0.0001
Late-life depression, assessed by self-reported diagnosis or treatment^3^						
Total flavonoids	1.00 (referent)	0.90 (0.81, 1.00)	0.91 (0.81, 1.01)	0.91 (0.82, 1.01)	0.87 (0.78, 0.97)	0.05
Flavonols	1.00 (referent)	1.10 (0.99, 1.21)	0.97 (0.87, 1.08)	0.96 (0.86, 1.07)	0.93 (0.84, 1.04)	0.03
Flavones	1.00 (referent)	1.04 (0.94, 1.16)	1.07 (0.96, 1.19)	0.99 (0.89, 1.11)	0.94 (0.84, 1.05)	0.14
Flavanones	1.00 (referent)	0.98 (0.88, 1.09)	1.02 (0.92, 1.14)	0.96 (0.86, 1.07)	0.91 (0.82, 1.02)	0.08
Flavan-3-ols	1.00 (referent)	0.95 (0.86, 1.05)	0.95 (0.86, 1.06)	0.87 (0.78, 0.97)	0.91 (0.82, 1.02)	0.11
Anthocyanins	1.00 (referent)	0.93 (0.84, 1.04)	0.96 (0.86, 1.06)	0.96 (0.86, 1.07)	1.04 (0.93, 1.16)	0.22
Polymeric flavonoids	1.00 (referent)	0.99 (0.89, 1.10)	0.96 (0.86, 1.07)	0.93 (0.84, 1.04)	0.88 (0.79, 0.99)	0.01
Proanthocyanidins	1.00 (referent)	0.92 (0.83, 1.03)	0.99 (0.89, 1.10)	0.96 (0.87, 1.07)	0.88 (0.78, 0.98)	0.05

1 *n* = 41,920. HRs (95% CIs) for depression by intake category of total flavonoids and individual subclasses of flavonoids were analyzed by using Cox proportional hazards models. The test for trend was performed by using the median value for each intake category. Model adjusted for age, questionnaire cycle, total energy intake (continuous), social network (quintiles), alternate Mediterranean diet score (quintiles; excluding components of vegetables, fruit, and alcohol), census-tract family income (<$50,000, $50,000–69,999, or ≥$70,000/y), alcohol intake (0, 0.1–4.9, 5.0–29.9, 30–44.9, or ≥45.0 g/d), subjective self-rated societal position (low or other), cigarette smoking (never; past; current: 1–14, 15–24, or ≥25 cigarettes/d), BMI (continuous), physical activity in total metabolic equivalents per week (continuous), actual sleep hours (≤6, 7–8, or ≥9 h/d), frequency of difficulty falling or staying asleep (none, little, or some to all of the time), bodily pain (none to mild or moderate to very severe), current multivitamin use (yes or no), coffee consumption (≤1 serving/wk, 2–6 servings/wk, 1 serving/d, 2–3 servings/d, or ≥4 servings/d), menopausal status and postmenopausal hormone use [premenopausal or postmenopausal (never postmenopausal hormone user, past postmenopausal hormone user, or current postmenopausal hormone user)], history of hypertension (yes or no), history of cardiovascular disease (yes or no), history of diabetes (yes or no), and history of hypercholesterolemia (yes or no). NHS, Nurses’ Health Study.

2 Late-life depression assessed by either self-reported physician diagnosis, regular use of antidepressant medication, or the presence of severe depressive symptoms. The incident rate was 6734 cases/278,582.4 = 24.2 cases/1000 person-years.

3 Late-life depression assessed by either self-reported physician diagnosis or regular use of antidepressant medication. The incident rate was 3527 cases/294,800 = 12.0 cases/1000 person-years.

**TABLE 5:** HRs (95% CIs) for associations between intakes of flavonoid-rich food and incident late-life depression among women aged ≥65 y in the NHS^1^

	Intake category^2^					
	1	2	3	4	5	*P*-trend
Citrus fruit and juices	1.00 (referent)	0.90 (0.75, 1.09)	0.88 (0.76, 1.01)	0.90 (0.77, 1.05)	0.84 (0.72, 0.97)	0.02
Tea	1.00 (referent)	1.02 (0.94, 1.11)	0.96 (0.89, 1.04)	0.98 (0.90, 1.07)	0.88 (0.75, 1.04)	0.11
Onions	1.00 (referent)	0.98 (0.89, 1.06)	1.01 (0.92, 1.10)	0.96 (0.88, 1.05)	0.93 (0.82, 1.04)	0.22
Apples or pears	1.00 (referent)	0.99 (0.83, 1.18)	0.91 (0.76, 1.09)	0.92 (0.77, 1.09)	0.84 (0.71, 1.01)	0.002
Strawberries	1.00 (referent)	0.99 (0.87, 1.12)	0.97 (0.85, 1.09)	0.95 (0.84, 1.08)	0.92 (0.73, 1.14)	0.22
Blueberries	1.00 (referent)	0.96 (0.90, 1.02)	0.95 (0.88, 1.02)	0.97 (0.88, 1.08)	0.70 (0.46, 1.07)	0.17
Red wine	1.00 (referent)	1.03 (0.96, 1.10)	0.98 (0.90, 1.08)	0.91 (0.83, 1.01)	0.94 (0.81, 1.08)	0.09

1 *n* = 41,920. HRs (95% CIs) for depression by intake category of total flavonoids and individual subclasses of flavonoids were analyzed by using Cox proportional hazards models. The test for trend was performed by using the median value for each intake category. Model adjusted for age, questionnaire cycle, total energy intake (continuous), social network (quintiles), alternate Mediterranean diet score (quintiles), census-tract family income (<$50,000, $50,000–69,999, or ≥$70,000/y), alcohol intake (0, 0.1–4.9, 5.0–29.9, 30–44.9, or ≥45.0 g/d), subjective self-rated societal position (low or other), cigarette smoking (never; past; current: 1–14, 15–24, or ≥25 cigarettes/d), BMI (continuous), physical activity in total metabolic equivalents per week (continuous), actual sleep hours (≤6, 7–8, or ≥9 h/d), frequency of difficulty falling or staying asleep (none, little, or some to all of the time), bodily pain (none to mild or moderate to very severe), current multivitamin use (yes or no), coffee consumption (≤1 serving/wk, 2–6 servings/wk, 1 serving/d, 2–3 servings/d, or ≥4 servings/d), menopausal status and postmenopausal hormone use [premenopausal or postmenopausal (never postmenopausal hormone user, past postmenopausal hormone user, or current postmenopausal hormone user)], history of hypertension (yes or no), history of cardiovascular disease (yes or no), history of diabetes (yes or no), and history of hypercholesterolemia (yes or no). Late-life depression was assessed by either self-reported physician diagnosis, regular use of antidepressant medication, or the presence of severe depressive symptoms. NHS, Nurses’ Health Study.

2 Category values were as follows—citrus fruit and juices: 1 (<1 serving/wk), 2 (1 serving/wk), 3 (2–4 servings/wk), 4 (5–6 servings/wk), and 5 (≥1 serving/d); tea: 1 (<1 serving/mo), 2 (≥1 serving/mo to ≤1 serving/wk), 3 (2–6 servings/wk), 4 (1–3 servings/d), and 5 (≥4 servings/d); onion, apples or pears, strawberries, blueberries, and red wine: 1 (<1 serving/mo), 2 (1–3 servings/mo), 3 (1 serving/wk), 4 (2–4 servings/wk), and 5 (≥5 servings/wk). One serving = 1 orange, one-half grapefruit, 1 small glass of orange juice, 1 small glass of grapefruit juice, 1 fresh apple or pear, 1 cup (240 mL) tea, 0.5 cup onions, 0.5 cup strawberries or blueberries, or one 4-ounce (120-mL) glass red wine.

## DISCUSSION

To our knowledge, this is the first large-scale prospective study to examine the association between self-reported habitual intakes of major flavonoid subclasses and depression risk. Combining via meta-analysis the results from the NHS and NHSII cohorts, which were composed of middle-aged (i.e., NHSII: mean age of 46 y at baseline) and older (i.e., NHS: mean age of 67 y at baseline) women, we observed that the highest intakes of flavonols, flavones, and flavanones were significantly associated with a 7–10% lower risk of depression compared with the lowest intakes; higher intakes of flavan-3-ols and anthocyanins were not significantly associated with lower depression risk. In addition, total flavonoids, polymeric flavonoids, and proanthocyanidins were associated only with a significant reduction in depression risk in the older cohort of participants (i.e., NHS) but not among the middle-aged women of the NHSII.

Although the association estimates were moderate and intervention trials are needed to further understand the clinical importance of these findings, several points can be highlighted. First, despite modest effect estimates, at a population level the results may be important. By using a population-attributable risk framework, 5% of the depression cases that occurred in both the NHS and NHSII could have been prevented if all women in the lowest 3 quintiles of flavanone intakes switched to the highest 2 quintile of intakes. Importantly, such computations require assumptions of causal links, which can only be established in randomized trials or other experimental approaches; thus, population-attributable risk estimates are more speculative in observational studies. Nevertheless, they are helpful at conveying the broader concept that small effect estimates from a common exposure can translate to relatively larger impacts on health. Second, although the magnitudes of overall estimates of association were modest in the primary analysis, the associations were stronger with respect to late-life depression. For example, women in the highest quintile of flavones and proanthocyanidins showed a significant 17% reduction in the risk of late-life depression. Third, the significant dose-response relation and consistency across 2 cohorts for several specific flavonoids made the observed inverse associations less likely to be due to chance.

Available evidence with regard to flavonoid-depression associations is limited, particularly data from epidemiologic studies and trials. Existing work has focused on intakes of a specific flavonoid-rich food (typically, cocoa or tea) or overall fruit and vegetable consumption. Inverse associations were observed in some studies, but most were cross-sectional and did not involve comprehensive evaluations of flavonoid subclasses or a broad range of food sources (36–38). Moreover, our results are in line with evidence of favorable relations of flavonoids to other later-life brain outcomes, including cognitive decline and Parkinson disease (13, 17). Finally, preliminary evidence from randomized controlled trials suggested benefits of specific flavonoid compounds for reducing depressive symptoms or enhancing mood (39, 40). However, most of these randomized controlled trials focused on short-term action of flavonoids on change in depressive symptoms and had small sample sizes. Overall, there is no comparable evidence with regard to whether long-term habitual intakes of flavonoids affect depression risk. Thus, the current study makes a unique contribution to the literature.

Although the specific links between flavonoids and depression are unclear, growing evidence supports a beneficial role for flavonoids on mood and brain health (41, 42), and direct and indirect mechanisms have been suggested (43). Direct mechanisms may include modulating signaling pathways responsible for maintaining neuron survival and inducing synaptic plasticity (7, 42, 44). Indirect mechanisms may include reducing neuroinflammation, improving blood flow, or reducing oxidative stress (6, 8, 9, 45). However, it is unclear whether the different flavonoid subclasses and their metabolites share generic neuroprotection properties or whether particular flavonoids are more neuroprotective than others. It is important to note that, although many flavonoid metabolites are shown to cross the blood-brain barrier (46, 47), the beneficial effects of specific flavonoids may depend on their bioavailability—influenced by absorption, metabolism, and disposition in tissues and cells—which differs greatly by subclasses (48, 49). For example, flavanones are more efficiently absorbed than most other subclasses and have longer elimination half-lives (50). Among the older cohort (i.e., NHS), polymers including proanthocyanidins were also associated with lower depression risk. Although the full range of biological mechanisms of flavonoids involved in depression remains to be elucidated, proanthocyanidin-rich botanical extracts have been shown to prevent depressive-like behaviors in proneurotoxin-treated rats and to promote anti-inflammatory and antioxidant activities in rats (51, 52).

Strengths of this study include the following: a large sample size; its prospective design; a comprehensive assessment of the full range of flavonoid subclasses in habitual diet; long-term, detailed, and repeated measures of flavonoids and covariates; and lengthy follow-up. Limitations also warrant discussion. For example, it is impossible to include every single food and nutrient in the FFQ, and flavonoid values calculated from the most recent databases (11) could be misclassified because flavonoid contents of foods vary by growth and processing conditions (53) and analytic extraction methods (54). In addition, some foods with different flavonoid contents are grouped together into single questions (e.g., apples and pears are grouped together, and black and green teas are not differentiated in the NHS/NHSII FFQs). Therefore, some degree of exposure misclassification is likely. However, the quartile ranking is less likely to be affected by such misclassification, and fruit and vegetable intakes assessed from an FFQ and weighted diet records correlated well in reproducibility and validity studies in the NHS and NHSII cohorts (19–21). Although we did not have objective biomarkers of flavonoid intakes, such as urinary metabolite excretion, measured in this study, good correlations between flavonoid biomarker concentrations and fruit/vegetable intakes (0.43–0.66) have been reported elsewhere (55). Potential misclassification of the depression outcome must also be considered, despite past success in using this definition to show important disease associations (26). For example, some degree of underascertainment of depression in this study is likely, because participants’ physicians or clinicians may not detect depression when it is present, clinicians may not inform participants of the depression diagnosis, or participants may elect not to report on the questionnaires the depression diagnoses they received or their antidepressant use information. However, the rates we observed are consistent with previously reported rates for major depression in women (34, 35). The rate observed for the broader depression definition in older women (diagnosis, treatment, and/or symptoms) is almost identical to that reported elsewhere when depression is similarly defined (34, 35). The use of the more conservative, clinical indicators-only outcome definition may bias results toward the null; therefore, our primary analysis results may be underestimates. In addition, SSRIs are prescribed not only for depression but also for other psychiatric conditions; this may generate false-positive depression cases. Some true depression cases may also have been missed because regular use of TCAs (<3% in both the NHS and NHSII) was not included in the outcome definition. However, the probability of correctly classifying individuals as cases is likely to be independent of flavonoid intake and thus should not bias the observed associations. Indeed, the results from the sensitivity analyses applying more or less strict definitions showed similar findings.

Other potential methodologic limitations should be discussed. First, despite careful adjustment for potential confounders, residual confounding is possible. Nevertheless, the detailed, updated adjustment for covariates render residual confounding unlikely to account fully for the findings. We also cannot rule out that other dietary components in fruit and vegetables may affect the results. However, further adjustment for omega-3 fatty acid and vitamin C, B-6, and B-12 or folate intakes did not attenuate the associations; in addition, the results remained the same when we adjusted for the aMed score in the model (which includes fruit and vegetable components). Second, our results could be biased if the censoring mechanism was dependent on the mechanism that produced the outcome of interest. However, follow-up was ∼90% in each 2-y cycle in both cohorts and the competing risks analysis showed similar results, suggesting that selection attrition was not a major concern. Finally, our findings from this all-female, predominantly white cohort may not be directly generalizable to men or to other race/ethnic groups. Further research to observe directly the relations between men and diverse race/ethnic groups would be valuable.

In these large prospective female cohorts with 10 y of follow-up, greater intakes of dietary flavonoids were significantly associated with a modest reduction in depression risk, particularly among older women. Associations varied by different flavonoid subclasses, pinpointing the importance of future studies to confirm comparable findings in other cohorts and to clarify the biological mechanisms whereby different flavonoid subclasses may contribute to depression prevention. Further prospective studies and intervention trials are needed to confirm these associations. If confirmed, the findings may have important implications for depression prevention, because there are limited, readily-modifiable risk factors for depression.

## Footnotes

9 Abbreviations used: aMed, alternate Mediterranean diet; CESD-10, 10-item version of the Center for Epidemiologic Studies Depression; FFQ, food-frequency questionnaire; GDS-15, 15-item version of the Geriatric Depression Scale; MHI-5, 5-item Mental Health Index; NHS, Nurses’ Health Study; NHSII, Nurses’ Health Study II; SSRI, selective serotonin reuptake inhibitor; TCA, tricyclic antidepressant.
